# Non-typhoidal *Salmonella* DNA traces in gallbladder cancer

**DOI:** 10.1186/s13027-016-0057-x

**Published:** 2016-03-03

**Authors:** Prajish Iyer, Savio George Barreto, Bikram Sahoo, Pratik Chandrani, Mukta R. Ramadwar, Shailesh V. Shrikhande, Amit Dutt

**Affiliations:** Advanced Centre for Treatment, Research and Education in Cancer, Kharghar, Navi Mumbai India; Department of Gastrointestinal Surgery, Gastrointestinal Oncology, and Bariatric Surgery, Medanta Institute of Digestive and Hepatobiliary Sciences, Medanta, The Medicity, Gurgaon, India; Department of Pathology, Tata Memorial Centre, Ernest Borges Marg, Parel, Mumbai, India; Department of Gastrointestinal and Hepato-Pancreato-Biliary Surgical Oncology, Tata Memorial Centre, Ernest Borges Marg, Parel, Mumbai, India

**Keywords:** *Salmonella*, Gall bladder cancer, Non-typhoidal, Inflammation

## Abstract

**Background:**

We earlier proposed a genetic model for gallbladder carcinogenesis and its dissemination cascade. However, the association of gallbladder cancer and ‘inflammatory stimulus’ to drive the initial cascade in the model remained unclear. A recent study suggested infection with *Salmonella* can lead to changes in the host signalling pathways in gallbladder cancer.

**Findings:**

We examined the whole exomes of 26 primary gall bladder tumour and paired normal samples for presence of 143 HPV (Human papilloma virus) types along with 6 common *Salmonella* serotypes (*S. typhi* Ty2, *S. typhi* CT18, *S. typhimurium* LT2, *S. choleraesuis* SCB67, S. *paratyphi* TCC, and *S. paratyphi* SPB7) using a computational subtraction pipeline based on the HPVDetector, we recently described. Based on our evaluation of 26 whole exome gallbladder primary tumours and matched normal samples: association of typhoidal *Salmonella* species were found in 11 of 26 gallbladder cancer samples, and non-typhoidal *Salmonella* species in 12 of 26 gallbladder cancer, with 6 samples were found co-infected with both.

**Conclusions:**

We present the first evidence to support the association of non-typhoidal *Salmonella* species along with typhoidal strains in gallbladder cancer. *Salmonella* infection in the chronic carrier state fits the role of the ‘inflammatory stimulus’ in the genetic model for gallbladder carcinogenesis that may play a role in gallbladder cancer analogous to *Helicobacter pylori* in gastric cancer.

**Electronic supplementary material:**

The online version of this article (doi:10.1186/s13027-016-0057-x) contains supplementary material, which is available to authorized users.

## Background

Gallbladder cancer with its attendant poor prognosis [1], despite the performance of good surgery when feasible [2], lends itself to the need for further research. Scanu et al. recently provided a novel mechanism by which chronic *Salmonella* infection of the gallbladder can lead to changes in the host signalling pathways triggering cellular transformations resembling the evolution of gallbladder cancer [3]. This is consistent with earlier epidemiological findings that support the association of typhoidal *Salmonella typhi* and *S.* paratyphi with the risk of gallbladder cancer [4–7]. However, non typhoidal species (*S. typhimurium*, *S choleraesuis*) that elicits a stronger host immune response compared to the typhoidal species and are linked to a systemic illness have as yet not reported to be associated with gallbladder disease or cancer [8]. Interestingly, in the Indian subcontinent where gallbladder incidence rates are amongst the highest in the world, *Salmonella* infection is also considerably severe [9].

We have previously proposed a model for gallbladder carcinogenesis based on the current understanding of the tumour biology and associated inflammation [10]. However, there remain lacunae in the model that need to be clarified. For instance, what is the ‘inflammatory stimulus’ that drives the initial cascade of an upregulation of inflammatory markers that is characterised by an increase in protective mucins as well as a strange divergence of inflammatory markers thereafter from the stage of *in situ* to invasive cancer? Here in this study we examined the exomes of primary gall bladder tumour and paired normal samples for presence of 6 common *Salmonella* serotypes with available genome information (*S. typhi* Ty2, *S. typhi* CT18, *S. typhimurium* LT2, *S. choleraesuis* SCB67, S. *paratyphi* TCC, and *S. paratyphi* SPB7) using a computational subtraction pipeline based on the HPVDetector tool [11]

## Methods

### Patient sample collection

Twenty six fresh frozen primary tumour and matched normal tissues were obtained from the tissue repository of Tata Memorial hospital (TMH). The Institutional Review Board (IRB) and the Ethics Committee (EC) of Tata Memorial Centre (TMC) - Advanced Centre for Treatment, Research and Education in Cancer (ACTREC) (Mumbai, India) approved the project (#104). Since this was a retrospective analysis, the IRB and the EC waived the need for an informed consent. Patients were randomly selected based on the availability of fresh frozen tissues. The patient characteristics including age, gender, gall stone status and histopathology were recorded.

### PCR analysis for *Salmonella* isolates

The PCR method used for *Salmonella* detection has been previously described [12]. Nested PCR was carried out in a 25 μl volume containing 10 μl KAPA 2X ready mix master-mix (KapaBiosystems catalog no-KK1024), 10 pmol primer and 100 ng of genomic DNA. Following the first round of PCR (94 °C for 1 min, 55 °C for 1 min 15 s, 72 °C for 3 min – 40 cycles) with ST1 and ST2 primers, 5 μl PCR product was used as template for nested PCR using ST3 and ST4 primers (94 °C for 1 min, 68 °C for 1 min 15 s, 72 °C for 3 min – 40 cycles). We also performed validation of *Salmonella* sequences using read specific primers. The PCR conditions - 94 °C for 1 min, 59 °C for 30s, 72 °C for 45 s – for 30 cycles. The primer sequences are detailed in Additional file 1: Table S2.

### Sequencing and analysis

Exome capture and library preparation was performed using Agilent Sure select in-solution (low-input capture-500 ng) target enrichment technology. Genomic DNA was sheared and size selected (150–200 bp) and ligated to adaptors and run on Illumina Nextseq 500 platform to generate 150 bp paired end reads at a coverage of 100X and above. To detect *Salmonella* traces, the HPVDetector pipeline was used, as described previously [11]. Briefly, reads were aligned against six known *Salmonella* species genomes in addition of the HPVDetector nce data set of 143 HPV types, as downloaded from the National Centre for Biotechnology Information (NCBI), using BWA (Burrows wheeler algorithm) aligner (v0.6.2). All reference sequences were annotated and concatenated to compose a multi-fasta sequences using bio-perl modules. The alignment files were parsed using UNIX shell program to detect the types of *Salmonella* represented by at least one read that aligned to a particular *Salmonella* type with high confidence.

## Results

### HPVDetector pipeline identifies *Salmonella* sequence present in gallbladder cancer samples

**Fig. 1:**
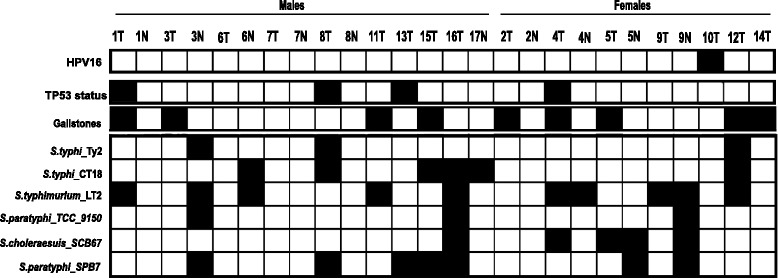
Profiling the occurrence of 143 HPV types and 6 *Salmonella* isolates across 26 gall bladder cancer patients. Heat map representation of 6 *Salmonella* isolates (in row) found across 26 gallbladder samples -- 17 tumours and 9 matched normal (in column) is shown. Solid boxes indicate presence of reads from *Salmonella* genome in the corresponding gallbladder sample. The samples (column) have been grouped based on gender as shown above the heat map. Solid boxes in the first row indicate presence of HPV16 sequence. Solid boxes in the second row indicate the presence of mutation in *TP53*. Reads of HPV16 were found in 1 of 26 samples (10 T). 9 of 17 gallbladder tumour samples were associated with gall stones as shown by solid boxes in the second row. Typhoidal *Salmonella* isolates were found in 11 of 26 gallbladder cancer samples, non-typhoidal *Salmonella* isolates were found in 12 of 26 gallbladder cancer, with 6 of 26 samples co-infected with both

We performed PCR based analysis of 26 gallbladder tumour and paired normal samples to detect the presence of *Salmonella* DNA using pan primers, as described earlier [12]. None of the gallbladder samples were found to be positive for *Salmonella* (data not shown). As a next step, whole exome data for these 26 samples (generated in house, manuscript in make) were analysed to detect *Salmonella* traces using HPVDetector pipeline, modified to include additional genome sequence of 6 common *Salmonella* isolates. The computational approach, in brief, subtracts all reads that align to human genome and aligns remaining reads to HPV and *Salmonella* reference database from NCBI. While HPV16 was detected in 1 gallbladder sample, *Salmonella* isolates were found across multiple samples: *S. typhi* Ty2 (3 samples), *S. typhi* CT18 (6 samples), *S. typhimurium* LT2 (10 samples), *S. choleraesuis* SCB67 (5 samples), *S. paratyphi* TCC (3 samples), and *S. paratyphi* SPB7 (7 samples). In total, *Salmonella* reads were found in 19 of 26 gallbladder tissues (tumor as well as adjacent normal tissues). Interestingly, 10 of 19 samples were co-infected with multiple isolates independent of gallstone status or gender of the patients, as shown in Fig. 1.

### Annotation of the *Salmonella* reads found in gallbladder cancer samples

Variable number of overlapping reads of variant lengths for each isolate were assembled into contigs based on Clustal *X*2 multiple alignment as shown in Additional file 2: Figure S1. The unique stretch of contigs generated were annotated based on gene annotation database of *Salmonella* isolates from NCBI (National centre for biotechnology information) database. 114 reads of multiple *Salmonella* isolates were found in 19 of 26 samples analyzed. 47 of 114 reads of *Salmonella* ORF (open reading frame) were identified as encoding for bacterium genes known to be involved in metabolism and those related to the toxin-antitoxin system. Rest of the reads aligned to the *Salmonella* ribosomal genes, understandably due to their relatively higher abundance (Additional file 3: Table S1).

### HPVDetector pipeline is specific and highly sensitive to detect true *Salmonella* traces

To assess specificity of our assay, we re-analyzed whole exome data of all samples by taking their reverse (not complement) to simulate random sequence, but retaining composition of nucleotides and genome complexity, using an in-house perl script, as described earlier [11]. We found no spurious *Salmonella* reads when the primary tumour whole exome sequence was reversed in any of the 26 samples, suggesting the computational pipeline used was specific to detect *Salmonella* traces. (Additional file 4: Figure S2A). To test the sensitivity of our assay, raw FASTQ file of a primary tumour sample 16 T that was found positive for *Salmonella* reads was down-sampled to 1X, 5X, 10X, 15X, 25X, 50X, 75X and 100X coverage using Picard Toolkit’s DownsampleSam function (http://broadinstitute.github.io/picard/), as described earlier [11]. The resulting FASTQ files were analysed for detection of *Salmonella* reads using the HPVDetector pipeline. Distinct *Salmonella* reads were detected at as low as 10X whole exome coverage that increased linearly (Additional file 4: Figure S2B).

### Sanger validation of *Salmonella* reads identified in gallbladder cancer samples

We have attempted to validate the presence of *Salmonella* read sequences identified by HPVDetector in 4 of 16 *Salmonella* positive samples using Sanger sequencing (Additional file 5: Figure S3).

## Discussion

We examined *Salmonella* and HPV DNA sequences in gallbladder tumours and paired normals, The high incidence of *Salmonella* sequence found in 16 of 26 samples analysed in the study suggests a possible role of *Salmonella* infection in gallbladder cancer analogous to *Helicobacter pylori* in gastric cancer and *Fusobacterium* in colon cancer [13, 14]. We demonstrate the presence of typhoidal *Salmonella* species in 11 of 26 gallbladder cancer samples, consistent with as known earlier. In addition, we present the first evidence to support the association of even non-typhoidal *Salmonella* species in 12 of 26 gallbladder cancer, with 6 of 26 samples co-infected with typhoidal as well as non typhoidal *Salmonella* isolates.

Systemic inflammation is known to be associated with a poor prognosis in gallbladder cancer [15]. Owing to the ability of *Salmonella* infection to stimulate a host response, it is likely that these bacteria are able to provide the continued ‘inflammatory stimulus’ necessary for carcinogenesis. Recent reports suggest that *Salmonella* infections promote malignant transformation in genetically predisposed mice, murine gall bladder organoids and fibroblasts with *TP53* mutations [3]. We observed 4 of 16 *Salmonella* positive samples harboured *TP53* mutations while we did not observe *TP53* mutations in *Salmonella* negative samples. *Salmonella* isolates in the chronic carrier state thus fits the role of the ‘inflammatory stimulus’ in the genetic model for gallbladder carcinogenesis and its dissemination cascade, which may trigger transformation through chronic inflammation, but not for maintenance of tumourigenesis [10].

The focus of treatment in typhoid-endemic countries such as India has historically been solely on eliminating typhoidal *Salmonella* species often underestimating the contribution of the non-typhoidal isolates that show an inherent higher resistance to the standard antibiotics [8] resulting in their ability to lead to chronic carrier state in humans. The presence of non-typhoidal *Salmonella* species in our study highlights that in typhoid-, as well as gallbladder cancer-endemic countries such as India and other similar countries, efforts must be directed not only at treating typhoid fever, but also diagnosing and appropriately managing non-typhoidal *Salmonella* species. This simple approach could reduce the chronic carrier state of these species in humans, which by our hypothesis may be contributing to the inflammatory stimulus driving gallbladder carcinogenesis. Thus, this simple strategy may help reduce in the incidence of gallbladder cancer. While this study validates and extends the association of *Salmonella* with gall bladder carcinoma, further study is required to establish the causality of infection to the disease.

## Additional files

Additional file 1: Table S2. Primer sequences used for detection of *Salmonella (PDF 122 kb)*
Additional file 2: Figure S1. Abundance and annotation of *Salmonella* reads found across the 16 of 26 gall bladder cancer samples. Heat map representation of individual *Salmonella* reads (in rows) identified from 6 different isolates found across the 16 gall bladder cancer samples (in column) is shown. Variable length and number of overlapping reads, each of 150 bp obtained from paired end Illumina sequence for each isolate, were assembled into contigs based on Clustal *X*2 multiple alignment. The unique total length of contigs generated is shown in second column reflecting the total length of the gene covered in the study. The contigs generated were annotated based on gene annotation database of *Salmonella* isolates from NCBI database. A representative general class for all genes identified is shown in the third column. (PDF 11190 kb) Additional file 3: Table S1. Detailed annotation table of read sequences of different *Salmonella* species identified across gallbladder cancer patient samples. (PDF 348 kb) Additional file 4: Figure S2. Specificity and Sensitivity for detection of *Salmonella* reads in whole exome sequence of gallbladder samples. (A) Specificity for detection of *Salmonella* reads in whole exome sequence of gallbladder samples. Exome sequenced reads were reversed (not complement) to maintain the genome complexity and used an input file to detect random *Salmonella* reads. No *Salmonella* reads were found in the samples with reversed whole exome sequence. (B) Sensitivity for detection of *Salmonella* reads in gallbladder samples as a function of increasing genome sequence coverage. Gallbladder tumour sample 16 T with highest number of *Salmonella* reads was down-sampled to 1x, 5x, 10x, 15x, 25x, 50x, 75x and 100x. *Salmonella* reads were counted (black line) and plotted against increasing coverage of the genome on x-axis. (PDF 5395 kb) Additional file 5: Figure S3. Sanger validation of *Salmonella* read sequences in gall bladder cancer samples Individual read sequences were PCR amplified and Sanger sequencing trace of individual read sequence with their blast output is represented in the figure. (PDF 1437 kb)

## Abbreviations

HPV: human papilloma virus
NCBI: national centre for biotechnology information
ORF: open reading frame
PCR: polymerase chain reaction
BWA: Burrows wheeler aligner
