# Supplementary material for: Non-typhoidal Salmonella DNA traces in gallbladder cancer
Source: Infect Agent Cancer. 2016 Mar 3;11:12. doi: 10.1186/s13027-016-0057-x (PMC4776363; doi:10.1186/s13027-016-0057-x)
Supplement: Additional file 1: Table S2. — Primer sequences used for detection of Salmonella (PDF 122 kb) [file 13027_2016_57_MOESM1_ESM.pdf]

Primer		5'-3'
OAD_1275_S typhi F 1st rnd F	ST1	TATGCCGCTACATATGATGAG
OAD_1276_S typhi R 1st rnd R	ST2	TTAACGCAGTAAAGAGAG
OAD_1277_S typhi F 2nd rnd F	ST3	AGATGGTACTGGCGTTGCTC
OAD_1278_S typhi F 2nd rnd R	ST4	TGGAGACTTCGGTCG CGTAG

Read specific primers

OAD 1352 _Stymurium_9T_F	TCGACCAGTGAGCTATTACGC	
OAD1353 _Stymurium_9T_R	GCCAGCTAAGGTCCCAAAGT	
OAD1354_Stymurium_4T_F	TCTCTCAAGCGCCTTGGTAT	
OAD1355_Stymurium_4T_R	CGAGGCACTACTGTGCTGAA	
OAD1361_Styphi_15T_F	CCAGCTCGCGTACCTCTTTA	
OAD1362_Styphi_15T_R	ATACCGCCCAAGAGGTCATA	
OAD1359_Stymurium_1T_F	CCCACATCGTTTCCCACTTA	
OAD1360_Stymurium_1T_R	GCGAATTCCGGAGAATGTTA	
