# Supplementary material for: Non-typhoidal Salmonella DNA traces in gallbladder cancer
Source: Infect Agent Cancer. 2016 Mar 3;11:12. doi: 10.1186/s13027-016-0057-x (PMC4776363; doi:10.1186/s13027-016-0057-x)
Supplement: Additional file 2: Figure S1. — Abundance and annotation of Salmonella reads found across the 16 of 26 gall bladder cancer samples. Heat map representation of individual Salmonella reads (in rows) identified from 6 different isolates found across the 16 gall bladder cancer samples (in column) is shown. Variable length and number of overlapping reads, each of 150 bp obtained from paired end Illumina sequence for each isolate, were assembled into contigs based on Clustal X2 multiple alignment. The unique total length of contigs generated is shown in second column reflecting the total length of the gene covered in the study. The contigs generated were annotated based on gene annotation database of Salmonella isolates from NCBI database. A representative general class for all genes identified is shown in the third column. (PDF 11190 kb) [file 13027_2016_57_MOESM2_ESM.pdf]

Salmonella isolates		Gene Class	17N	8T	16T	12T	1T	11T	4T	5T	9N	15T	9T	13T	3N	4N	5N	6N
*S.typhi*_Ty2	158	23srRNA genes		1														
	151					1												
	151														1			
*S.typhi*_CT18	151	Intron			1													1
	151	23srRNA genes	1			1												
	158			1														
	151				1													
	151					1												
	185											1						
*S.typhimurium*_LT2	180	Intron			1													
	151	23srRNA genes<br>protein metabolism			1													
	161				1			1			1				1			
	204					1												
	151						1		1									
	174												1					
	151												1					
	207														1			
	157														1	1		
	166														1			
	190																	1
S.paratyphi TCC9150	151	Intron			1													
	151	Genes predicted to be involved in metabolism			1													
	151				1													
	151				1													
	151				1													
	152										1							
	152										1							
	151										1							
	151										1							
	151														1			
	205														1			
S.choleraesuis SCB67	331	Intron			1													
	169	Pseudogenes			1													
	151				1													
	151				1													
	151								1									
	151								1								1	
	152									1								
	151										1							
	173																1	
S.paratyphi_SPB7	158	Genes predicted to be involved in toxin-antitoxin system<br>23srRNA genes<br>16srRNA gene<br>oxidoreductases		1	1									1				
	153			1								1					1	
	158			1														
	208				1													
	180				1													
	151				1													
	151				1													
	151				1													
	156										1				1		1	
	151										1							
	157										1				1		1	
