# Supplementary figures and images for: Non-typhoidal Salmonella DNA traces in gallbladder cancer
Source: Infect Agent Cancer. 2016 Mar 3;11:12. doi: 10.1186/s13027-016-0057-x (PMC4776363; doi:10.1186/s13027-016-0057-x)

A

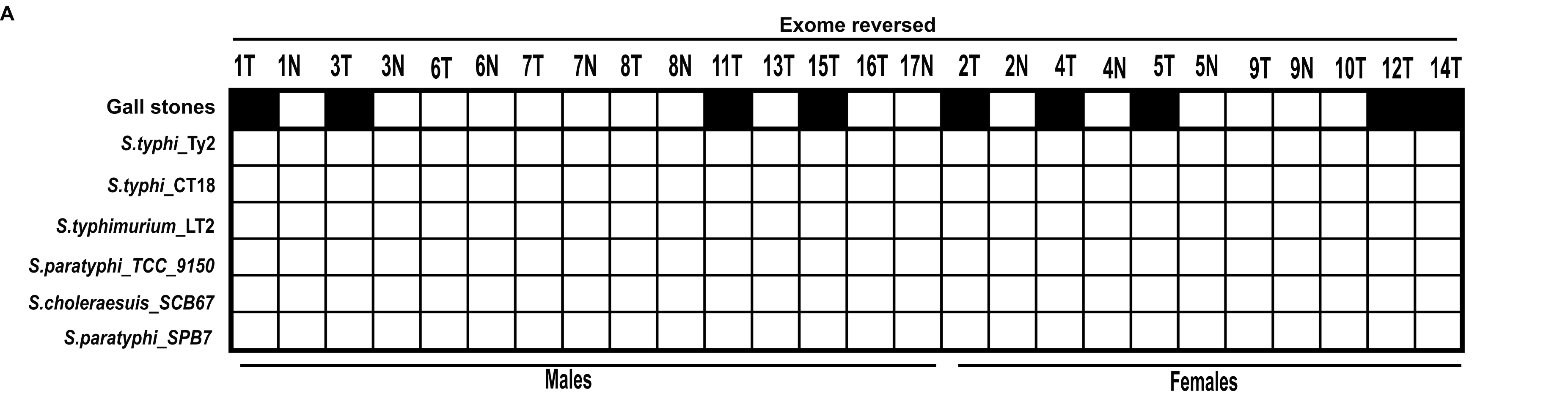

B

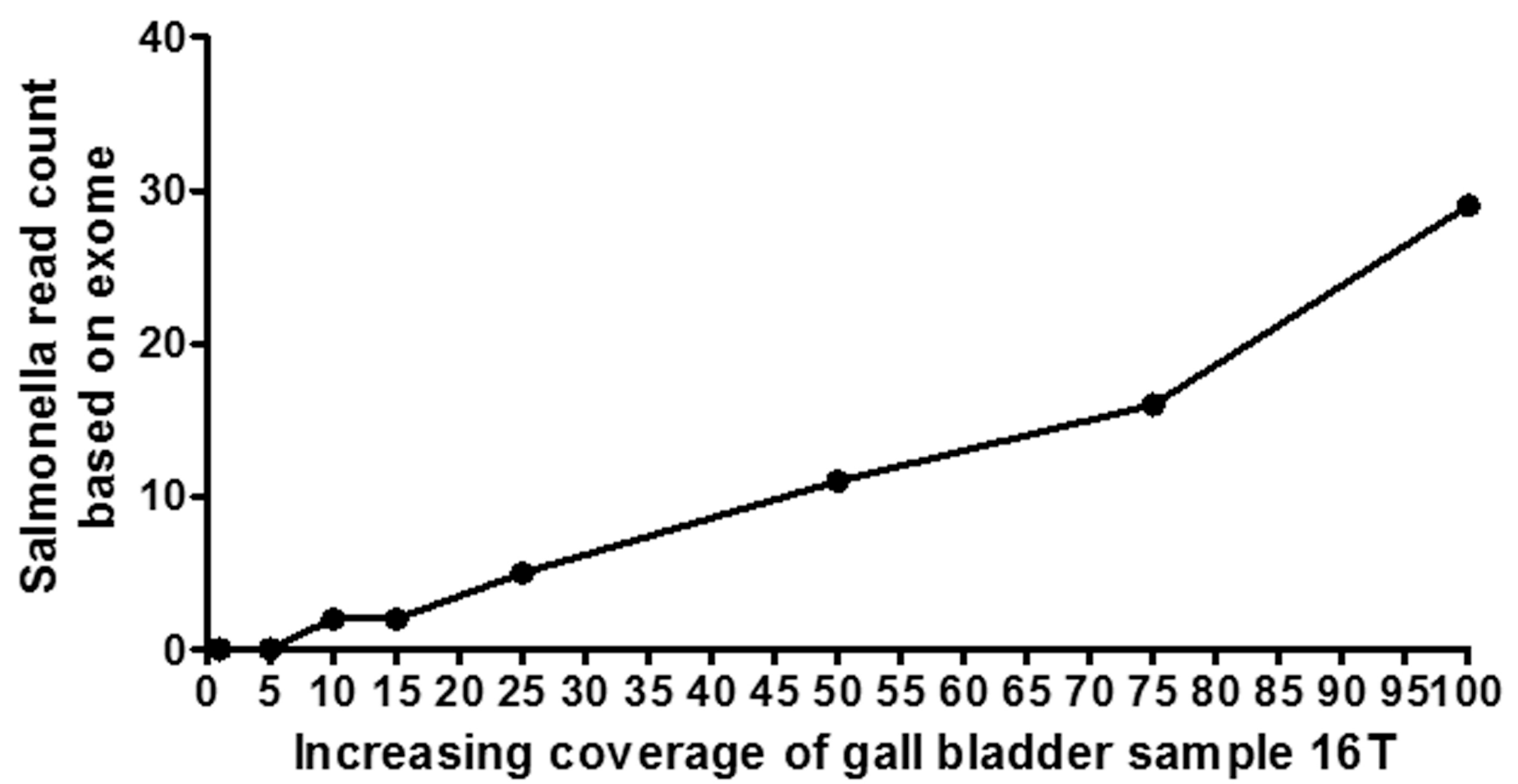

Supplement: Additional file 4: Figure S2. — Specificity and Sensitivity for detection of Salmonella reads in whole exome sequence of gallbladder samples. (A) Specificity for detection of Salmonella reads in whole exome sequence of gallbladder samples. Exome sequenced reads were reversed (not complement) to maintain the genome complexity and used an input file to detect random Salmonella reads. No Salmonella reads were found in the samples with reversed whole exome sequence. (B) Sensitivity for detection of Salmonella reads in gallbladder samples as a function of increasing genome sequence coverage. Gallbladder tumour sample 16 T with highest number of Salmonella reads was down-sampled to 1x, 5x, 10x, 15x, 25x, 50x, 75x and 100x. Salmonella reads were counted (black line) and plotted against increasing coverage of the genome on x-axis. (PDF 5395 kb) [file 13027_2016_57_MOESM4_ESM.pdf]
