# Supplementary material for: Non-typhoidal Salmonella DNA traces in gallbladder cancer
Source: Infect Agent Cancer. 2016 Mar 3;11:12. doi: 10.1186/s13027-016-0057-x (PMC4776363; doi:10.1186/s13027-016-0057-x)
Supplement: Additional file 5: Figure S3. — Sanger validation of Salmonella read sequences in gall bladder cancer samples Individual read sequences were PCR amplified and Sanger sequencing trace of individual read sequence with their blast output is represented in the figure. (PDF 1437 kb) [file 13027_2016_57_MOESM5_ESM.pdf]

Sample 15T

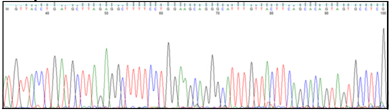

Salmonella enterica subsp. enterica serovar Choleraesuis strain C500, complete genome
Sequence ID: gb|CP007639.1| Length: 4751585 Number of Matches: 6

Range 1: 4093811 to 4093893 GenBank Graphics

Score	Expect	Identities	Gaps	Strand
137 bits(74)	3e-29	79/83(95%)	0/83(0%)	Plus/Minus

```
Query  15       TTGGGACTRCTTCAGCCCCAGGATGWGATGAGCCGRCATCGAGGTGCCAAACACCGCCGT  74
                |||||||| |||||||||||||||| ||||||||| ||||||||||||||||||||||||
Sbjct  4093893  TTGGGACTACTTCAGCCCCAGGATGTGATGAGCCGACATCGAGGTGCCAAACACCGCCGT  4093834

Query  75       CGATATGACCTCTTGGGCGGTAT  97
                |||||||| ||||||||||||||
Sbjct  4093833  CGATATGAACTCTTGGGCGGTAT  4093811
```

Sample 4T

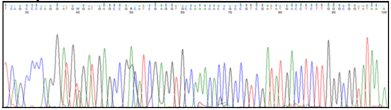

Salmonella enterica subsp. enterica serovar Pullorum str. ATCC 9120, complete genome
Sequence ID: gb|CP012347.1| Length: 4694842 Number of Matches: 7

Range 1: 465209 to 465297 GenBank Graphics ▼ Next Match ▲ Previous Match

Score	Expect	Identities	Gaps	Strand
128 bits(69)	2e-26	82/89(92%)	3/89(3%)	Plus/Minus

Features: rRNA-23S ribosomal RNA

```
Query  15      TCGG-TTGGGGT-MGATTT-RWGTTACCTGATGCTTAGAGGCTTTTCCTGGAAGCAGGGC  71
               |||| |||||||  |||||   ||||||||||||||||||||||||||||||||||||||
Sbjct  465297  TCGGTTTGGGGTACGATTTGATGTTACCTGATGCTTAGAGGCTTTTCCTGGAAGCAGGGC  465238

Query  72      ATTTGTTACTTCAGCACAGTAGTGCCTCG  100
               ||||||||||||||||| |||||||||||
Sbjct  465237  ATTTGTTACTTCAGCACCGTAGTGCCTCG  465209
```

Sample 9T

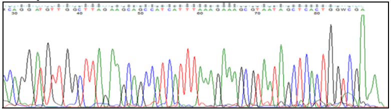

Salmonella enterica subsp. enterica serovar Bareilly str. CFSAN000189, complete genome
Sequence ID: gb|CP006053.1| Length: 4730612 Number of Matches: 7

Range 1: 524605 to 524686 GenBank Graphics ▼ Next Match ▲ Previous Match

Score	Expect	Identities	Gaps	Strand
126 bits(68)	4e-26	76/82(93%)	1/82(1%)	Plus/Plus

Features: rRNA-23S ribosomal RNA

```
Query  7       CGATGWGRG-AGGCAYAGACAGMCAGGATGTTGGCTTAGAAGCAGCCATCATTTAAAGAA  65
               ||||| | | ||||| |||||| |||||||||||||||||||||||||||||||||||||
Sbjct  524605  CGATGTGGGAAGGCACAGACAGCCAGGATGTTGGCTTAGAAGCAGCCATCATTTAAAGAA  524664

Query  66      AGCGTAATAGCTCACTGGWCGA  87
               |||||||||||||||||| |||
Sbjct  524665  AGCGTAATAGCTCACTGGTCGA  524686
```

Sample 1T

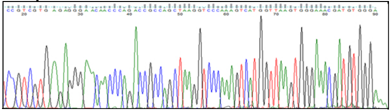

Salmonella enterica subsp. enterica serovar Paratyphi A str. AKU_12601 complete genome, strain AKU_12601
Sequence ID: emb|FM200053.1| Length: 4581797 Number of Matches: 7

Range 1: 3371134 to 3371222 GenBank Graphics

Score	Expect	Identities	Gaps	Strand
139 bits(75)	6e-30	85/89(96%)	4/89(4%)	Plus/Minus

Features: rRNA-23S ribosomal RNA

```
Query  6        CGA-TGCT-ACGTCCGTCGTGA-GAGGG-AACAACCCAGACCGCCAGCTAAGGTCCCAAA  61
                ||| |||| ||||||||||||| ||||| |||||||||||||||||||||||||||||||
Sbjct  3371222  CGAGTGCTAACGTCCGTCGTGAAGAGGGAAACAACCCAGACCGCCAGCTAAGGTCCCAAA  3371163

Query  62       GTCATGGTTAAGTGGGAAACGATGTGGGA  90
                |||||||||||||||||||||||||||||
Sbjct  3371162  GTCATGGTTAAGTGGGAAACGATGTGGGA  3371134
```
